# Immunophenotyping of Peripheral Blood, Lymph Node, and Bone Marrow T Lymphocytes During Canine Leishmaniosis and the Impact of Antileishmanial Chemotherapy

**DOI:** 10.3389/fvets.2020.00375

**Published:** 2020-07-15

**Authors:** Marcos Ferreira Santos, Graça Alexandre-Pires, Maria A. Pereira, Lídia Gomes, Armanda V. Rodrigues, Alexandra Basso, Ana Reisinho, José Meireles, Gabriela M. Santos-Gomes, Isabel Pereira da Fonseca

**Affiliations:** ^1^CIISA-Centro de Investigação Interdisciplinar em Sanidade Animal, Faculdade de Medicina Veterinária, Universidade de Lisboa, Lisbon, Portugal; ^2^GHTM-Global Health and Tropical Medicine (GHTM), Instituto de Higiene e Medicina Tropical (IHMT), Universidade Nova De Lisbon (UNL), Lisbon, Portugal

**Keywords:** antileishmanial therapy, bone marrow, canine leishmaniosis, effector T cells, flow cytometry, lymph node, peripheral blood mononuclear cells, regulatory T (Treg) cells

## Abstract

Dogs are a major reservoir of *Leishmania infantum*, etiological agent of canine leishmaniosis (CanL) a zoonotic visceral disease of worldwide concern. Therapeutic protocols based on antileishmanial drugs are commonly used to treat sick dogs and improve their clinical condition. To better understand the impact of *Leishmania* infection and antileishmanial drugs on the dog's immune response, this study investigates the profile of CD4^+^ and CD8^+^ T cell subsets in peripheral blood, lymph node, and bone marrow of sick dogs and after two different CanL treatments. Two CanL groups of six dogs each were treated with either miltefosine or meglumine antimoniate combined with allopurinol. Another group of 10 clinically healthy dogs was used as control. Upon diagnosis and during the following 3 months of treatment, peripheral blood, popliteal lymph node, and bone marrow mononuclear cells were collected, labeled for surface markers CD45, CD3, CD4, CD8, CD25, and intracellular nuclear factor FoxP3, and T lymphocyte subpopulations were immunophenotyped by flow cytometry. CanL dogs presented an overall increased frequency of CD8^+^ and CD4^+^CD8^+^ double-positive T cells in all tissues and a decreased frequency of CD4^+^ T cells in the blood. Furthermore, there was a higher frequency of CD8^+^ T cells expressing CD25^+^FoxP3^+^ in the blood and bone marrow. During treatment, these subsets recovered to levels similar to those of healthy dogs. Nevertheless, antileishmanial therapy caused an increase of CD4^+^CD25^+^FoxP3^+^ T cells in all tissues, associated with the decrease of CD8^+^CD25^−^FoxP3^−^ T cell percentages. These findings may support previous studies that indicate that *L. infantum* manipulates the dog's immune system to avoid the development of a protective response, ensuring the parasite's survival and the conditions that allow the completion of *Leishmania* life cycle. Both treatments used appear to have an effect on the dog's immune response, proving to be effective in promoting the normalization of T cell subsets.

## Introduction

Leishmaniosis is considered a neglected tropical disease (1) that affects humans and domestic and sylvatic animals. Parasites of the genus *Leishmania* are obligatory intracellular protozoa and the etiological agent of this parasitic disease (2). The main host cell for *Leishmania* parasites is the macrophage, which the parasite is able to manipulate and prevent activation by various mechanisms and, thus, avoid their intracellular death and perpetuate the infection (3–5). Canine leishmaniosis (CanL), endemic in about 50 countries and two major regions, South America and the Mediterranean basin, is caused by *Leishmania infantum* (6). Dogs affected by this disease can present a wide variety of specific and unspecific clinical signs (7, 8). CanL conventional treatments improve the clinical condition of dogs and reduce the parasite burden (9). Although when therapy is discontinued, relapses are common (10–12), indicating that treatment does not promote parasite clearance in all cases. Thus, it is important to improve the efficacy of the treatment protocols applied to CanL to promote the clinical cure of the dog, ensure parasite clearance, and prevent further transmission. According to the most recent guidelines (8), the recommended CanL treatment protocols combine allopurinol with either meglumine antimoniate or miltefosine. Meglumine antimoniate is a pentavalent antimonial considered a multifactorial drug whose effects are still unclear. However, some authors have referred the promotion of *Leishmania* DNA damage by oxidative stress and influence on macrophage microbicidal activity (13–15). Pentavalent antimonials, which belong to the same family of meglumine antimoniate, such as sodium antimony gluconate, have been shown to interfere with the host's immune system by activating macrophages to release interleukin 12 (IL-12), leading to the subsequent production of interferon-γ (IFN-γ) by other immune cells, that induce the phosphorylation of extracellular signal-regulated kinase 1 (ERK-1) and ERK-2, driving the production of reactive oxygen species (ROS) (16). Moreover, they also appear to induce the expression of class I molecules of the major histocompatibility complex (MHC), stimulating CD8^+^ T cells that lead to apoptosis of infected cells (17, 18). Although these drugs have proved antileishmanial activity *in vitro* and *in vivo*, pentavalent antimonials have failed to treat visceral leishmaniosis in human patients who are also infected with HIV or receiving immunosuppressive therapy (17), indicating that a complete cure is dependent on T cell-mediated responses (19, 20). Miltefosine is an alkylphosphocholine compound able to induce apoptosis by mechanisms still not entirely clear, although the specific disturbance of the lipid content on the parasite's membrane and the modulation of macrophage activity are the most consensual modes of action (18, 21–24). Several studies have reported the immunomodulatory properties of miltefosine, with *in vitro* studies showing the induction of the release of tumor necrosis factor α (TNF-α) and nitric oxide (NO) by peritoneal macrophages of BALB/c mice (25) and enhancement of IFN-γ receptors, thus restoring responsiveness to this cytokine in macrophages infected by *L. donovani* and promoting an IL-12-dependent Th1 response (26). Also, in healthy human peripheral blood cells, it was found that miltefosine was able to increase the production of IFN-γ, acting as a co-stimulator of the IL-2-mediated T cell activation process, together with increased expression of CD25, showing the possible immunomodulatory activity of miltefosine (27). Allopurinol, a purine analog of adenosine nucleotide, blocks RNA synthesis, inhibiting *Leishmania* growth (28, 29). To date, meglumine antimoniate or miltefosine in combination with allopurinol are both considered first-line treatments in Europe (7, 8). Recently, in Brazil, miltefosine therapy was approved for CanL treatment (30). Taking into account the emergence of a greater number of reports on drug resistance, whether it be in humans or dogs (13, 17, 21, 31), it is crucial to deepen the understanding of the mode of action of the most used antileishmanial therapies.

In dogs, disease outcome is mainly determined by the cell-mediated immune response, with T cells playing a key role in cytokine release, which interacts with infected macrophages, influencing macrophage activation and subsequent killing of internalized parasites. According to the cytokine environment, naive CD4^+^ T lymphocytes can differentiate into a protective subset (Th1) or a Th2 cell subset, which favors the progress of infection (32). A protective Th1 immune response is characterized by a high production of pro-inflammatory cytokines as is the case of IFN-γ, TNF-α, and IL-2. These cytokines stimulate the cytotoxic activity of CD8^+^ T cells and activate macrophage respiratory burst, leading to the synthesis of ROS and induce NO production, which can cause major damage to the parasite membrane, leading to the death of the parasite (32–34). On the other hand, a Th2 response directs the release of anti-inflammatory cytokines and stimulates the humoral immune response, favoring the establishment of infection and disease exacerbation (6, 7). Previous works on symptomatic dogs with CanL have demonstrated that the lack of adequate cell-mediated immune response might be associated with decreased levels of CD4^+^ T cells and high antibody titers (35–38). *In vitro* studies of cytotoxic CD8^+^ T cells from asymptomatic dogs demonstrated a role in resistance to CanL by enhancing IFN-γ production and causing the lysis of infected macrophages (39).

A critical role of immune regulation has been attributed to a subgroup of cells denominated regulatory T (Treg) cells, which seem to be recruited to the sites of *Leishmania* infection, enabling parasite survival and ensuring the transmission cycle (40, 41). Experimental studies of cutaneous leishmaniosis performed in *L. major*-infected mice showed that Treg cells are essential for the development and maintenance of persistent cutaneous disease (40). The fast increase of CD4^+^CD25^+^ Treg cells at the sites of *L. major* infection suppressed parasite-eliminating immune mechanisms (41). Accumulation of IL-10-producing Treg cells observed in the bone marrow of patients with *L. donovani* visceral leishmaniosis can cause immunosuppression, prevent the release of pro-inflammatory cytokines, like IFN-γ, avoid macrophage activation, and be associated with unresponsiveness to treatment (42). Another study showed increased CD4^+^CD25^+^ Treg cells exhibiting high levels of Forkhead box Protein 3 (FoxP3) gene expression along with transforming growth factor β (TGF-β) in spleen and draining lymph nodes of BALB/c mice infected with *L. infantum* (43). This cell subpopulation contributes to immunosuppression and control of parasite-mediated immunopathology during infection. Treg cell subsets that constitutively express CD25 and synthesize IL-10 and TGF-β drive the suppression of cell-mediated immune responses (44). These cells are considered potent suppressors of the activation of CD8^+^ T cells (45). Nevertheless, another study showed a reduced percentage of CD3^+^CD4^+^FoxP3^+^ Treg cells in dogs infected with *L. infantum*, independently of antibody titer (46). Although CD8^+^ T suppressor cells have been identified, their mode of action and purpose are not fully understood (47). Some studies have shown that resting CD4^+^ lymphocytes are resistant to CD8^+^CD25^+^FoxP3^+^ Treg cells, which indicates that the initiation of cell-mediated immune response is not likely to be affected by CD8^+^ Treg cells. In contrast, CD8^+^ Treg cells can play a critical role in suppressing ongoing CD4^+^ T cell responses (48). Besides, the activity of CD4^+^CD25^+^FoxP3^+^ Treg cells appears to be mediated through the release of immune-suppressive cytokines and by cell contact-dependent mechanisms (48). With regard to leishmaniosis, few studies focus on Treg cells, and less are those that have analyzed the CD8^+^ Treg cell fraction. Tiwananthagorn et al. (49) reported that in the liver of *L. donovani*-infected mice, CD4^+^FoxP3^+^ Treg cells, but not CD8^+^FoxP3^+^ T cells, are essential for the increased susceptibility to *Leishmania* infection and high IL-10 production.

T cells expressing both CD4 and CD8 molecules have been identified in peripheral blood and secondary lymphoid organs of several species, such as pigs, monkeys, humans, chickens, rats, mice, and dogs (50–56). These CD4^+^CD8^+^ double-positive (dp) T cells appear to constitute memory CD4^+^ helper T cells that, upon activation, develop the ability to express the CD8α chain and, in cases such as pigs, produce high levels of IFN-γ in response to stimulation with viral antigens (50). This subpopulation has been identified as being increased in chronic diseases, such as cancer, autoimmune diseases, and viral infections (57–61). Several studies have also reported the presence of CD25 and FoxP3 in dp T cells of dogs, revealing a possible regulatory activity among this subpopulation (62, 63).

Thus, the current study aims to evaluate the kinetics of CD4^+^ and CD8^+^ T cell subsets in tissues that commonly harbor *Leishmania* parasites in both sick and treated dogs. Sick dogs (CanL) were treated by two of the most used protocols for CanL during a 3-month period, and peripheral blood, lymph node, and bone marrow T cells were immunophenotyped.

## Materials and Methods

### Dog Selection

**Figure 1:**
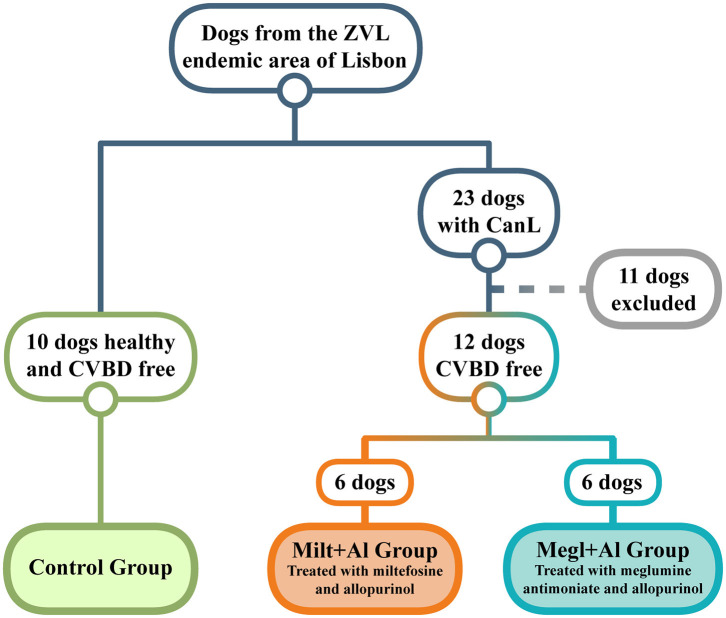
Dog selection diagram used in the current study. From a population of dogs living in an endemic area of zoonotic visceral leishmaniosis (ZVL), two groups clinically diagnosed with canine leishmaniosis (CanL) were established and treated with either miltefosine in combination with allopurinol (Milt+Al) or meglumine antimoniate in association with allopurinol (Megl+Al). A group of clinically healthy dogs and free of any canine vector-borne disease (CVBD) was also selected as the control group.

Twenty-three household dogs living in the endemic area of the Metropolitan Region of Lisbon (Portugal) were diagnosed with CanL at clinical stage I/II, according to the LeishVet Consensus Guidelines (64), and at stage C, following the Canine Leishmaniasis Working Group Guidelines (65). Twelve of these sick dogs fulfilled the minimum requirements to enter the study (Figure 1), which included having at least 1.5 years of age, weighing more than 5 kg, not having been vaccinated for leishmaniosis, being negative for circulating pathogens potentially responsible of canine vector-borne diseases (CVBDs), and have not undergone any treatment in the last 8 months that could interfere with the immune response (such as corticosteroids, antibiotics, or immunomodulators). The present study also included a control group of 10 clinically healthy dogs that were negative for *Leishmania* antibodies and other CVBDs and not vaccinated for leishmaniosis. All dog owners gave written consent after being informed about the objectives of the study and every procedure. The selected animals included 15 males and 7 females of various breeds, with ages ranging between 2 and 9 years and weight between 7.6 and 32.1 kg. Clinical examination and sample collection were done by veterinarians at the Teaching Hospital of the Faculty of Veterinary Medicine, University of Lisbon.

As previously described by our group (66), dogs diagnosed with CanL that presented biochemical parameters such as increased blood urea nitrogen (BUN), creatinine, and/or alanine aminotransferase (ALT), aspartate aminotransferase (AST), and urine protein-to-creatinine (UPC) ratio between 0.2 and 0.6, which point to the possibility of developing hepatic and renal lesions, were treated with miltefosine [Milteforan®, Virbac S.A., France; 2 mg/kg *per os, semel in die* (SID) for 4 weeks] combined with allopurinol [Zyloric®, Laboratórios Vitória, Portugal; 10 mg/kg, *per os, bis in die* (BID) for at least 6 months] and correspond to Group Milt+Al. Dogs that exhibited changes in serum proteins and UPC ratios between 0.2 and 0.4 were treated with meglumine antimoniate (Glucantime®, Merial Portuguesa, Portugal; 100 mg/kg SID for 4 weeks) combined with allopurinol (10 mg/kg, *per os*, BID for at least 6 months) and were included in Group Megl+Al. To prevent new infections during the study and *Leishmania* transmission, deltamethrin-impregnated collars were applied to all dogs.

### Experimental Design

To investigate the effect of *Leishmania* infection and antileishmanial treatments in helper, cytotoxic, and regulatory T cell subsets, peripheral blood, popliteal lymph node, and bone marrow mononuclear cells were isolated from sick dogs (CanL) before the beginning of treatment (M0) and monthly after treatment (M1, M2, and M3). These cells were immunophenotyped by evaluating the surface expression of CD45, CD3, CD4, CD8, and CD25 and the intracellular expression of FoxP3. To reduce the number of animals used in this study and to ensure any ethical concern for animal discomfort and well-being, the amount of sample collection and its periodicity were reduced to a minimum. Furthermore, peripheral blood, popliteal lymph node, and bone marrow samples were collected from sick dogs before the onset of treatment (M0) to establish the baseline levels of cell populations, avoiding the need of an additional group of untreated sick dogs. Peripheral blood, popliteal lymph node, and bone marrow samples were also collected from clinically healthy dogs [control group (CG)]. The present study followed the directive 86/609/EEC of the Council of the European Union and was approved by the Ethics and Animal Welfare Committee of the Faculty of Veterinary Medicine, University of Lisbon.

### Isolation of Peripheral Blood, Lymph Node, and Bone Marrow Mononuclear Cells

Peripheral blood mononuclear cells were obtained through density gradient centrifugation (Histopaque®-1077 solution, Sigma-Aldrich, Germany). Dog peripheral blood was resuspended in PBS (1:1 v/v), overlaid on half of that total volume in Histopaque®-1077 solution and centrifuged 400 × g for 30 min at 18°C. Peripheral blood mononuclear cells were then harvested at the interface of PBS and Histopaque® and washed twice in cold PBS (300 × g, 10 min, 4°C). Whenever red blood cells were still visible in the pellet, a step of lysis was done by adding 5 ml of RBC Lysis Buffer (eBioscience, USA) for 5 min and stopping the reaction with 10 ml of PBS, followed by a centrifugation at 300 × g (4°C) for 10 min. The pellet was then resuspended in Flow Cytometry Staining Buffer (FCSB) (eBioscience), and the total volume was adjusted for 2 × 10^7^ cells ml^−1^. Lymph node and bone marrow aspirates were centrifuged at 400 × g (4°C) for 5 and 15 min, respectively, and resuspended in FCSB with the total volume also adjusted for 2 × 10^7^ cells ml^−1^. These samples were then kept on ice until antibody labeling.

### Flow Cytometry

**Figure 2:**
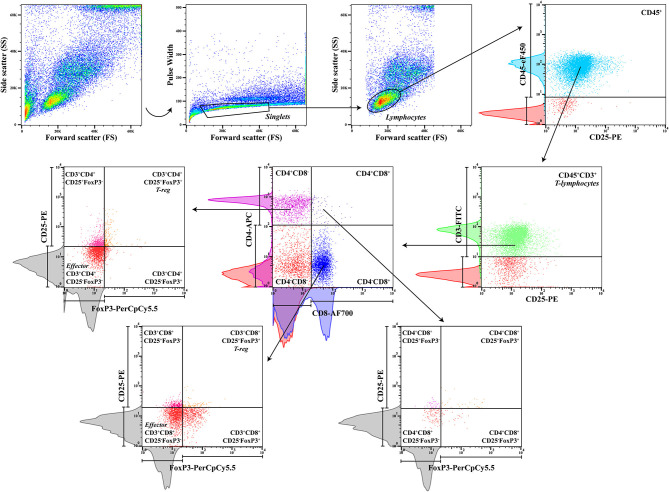
Gating strategy. Peripheral blood sequential gating strategy for a panel of six antibodies to identify the different cell subpopulations after doublet exclusion. CD45, a pan-leukocyte marker, and CD3, a T-lymphocyte specific marker, were used to define the T-lymphocyte population, with posterior separation of CD4^+^ and CD8^+^ cells, CD4^+^CD8^+^ double-positive T cells, and subsequent regulatory CD25^+^FoxP3^+^ and effector CD25^−^FoxP3^−^ cells. Red histograms from unstained control samples and colored histograms from single-stained control samples were used to define the sequential gating, along with gray histograms from fluorescence minus one (FMO) controls to gate for rare cells (CD25^+^FoxP3^+^).

To characterize regulatory and effector T cell subpopulations, a multicolor panel was designed for flow cytometry analysis, and each fluorochrome-conjugated antibody was titrated for optimal staining (Table 1). Cell suspensions (50 μl) were incubated with the following monoclonal antibodies (30 min at 4°C in the dark): rat anti-dog CD45 (clone YKIX716.13, eBioscience Inc.), mouse anti-dog CD3 (clone CA17.2A12, AbD Serotec, UK), anti-dog CD4 (clone YKIX302.9, eBioscience Inc.), rat anti-dog CD8 (clone YCATE55.9, AbD Serotec), and mouse anti-dog CD25 (clone P4A10, eBioscience Inc.) (Table 2). Then, cells were washed twice with 1 ml of FCSB and centrifuged at 400 × g (4°C) for 5 min. Afterward, 1 ml of FoxP3/Transcription Factor Fixation/Permeabilization Working Solution (eBioscience Inc.) was added, and cells were incubated overnight at 4°C in the dark. Next, 500 μl of 1× Permeabilization Buffer (eBioscience Inc.) was added, and cells were centrifuged at 400 × g (4°C) for 5 min, followed by two washes at 400 × g (4°C) for 5 min with 1 ml of 1× Permeabilization Buffer and a last washing step with 500 μl of FCSB. Cells were resuspended in a total of 100 μl of FCSB and incubated for 15 min at 4°C in the dark. Intracellular staining with anti-mouse/rat FoxP3 (clone FJK-16s, eBioscience Inc.) monoclonal antibody was done by incubating for at least 30 min (4°C) in the dark, followed by two washes with 1× Permeabilization Buffer at 400 × g (4°C) for 5 min. For flow cytometry acquisition (three-laser equipped CyAn ADP apparatus, Beckman Coulter, using the Summit v4.3, Dako Colorado Inc. software), cells were resuspended in a final volume of 300 μl of FCSB. For each sample, a minimum of 20,000 gated events were acquired, and data analysis was performed using FlowJo version 10.0.7 (Tree Star, CA). To define the best gating strategy to be applied (Figure 2), compensation was done with unstained, single-stained, and “fluorescence minus one” (FMO) samples (Table 2).

**Table 1:** Flow cytometer setup, fluorochrome panel, and labeling.

**Instrument: Beckman Coulter Cyan ADP**																														
Laser lines	405 nm					488 nm															642 nm									
Emission filters	450/50					530/40					575/25					680/30					665/20					750LP				
Fluorochrome	eFluor^®^ 450					FITC					PE					PerCP/Cy5.5					APC					Alexa Fluor^®^ 700				
Biomarker	CD45					CD3					CD25					FoxP3					CD4					CD8				
Brightness																														
Antibody	rat anti-dog					mouse anti-dog					mouse anti-dog					anti-mouse/rat					rat anti-dog					rat anti-dog				
Clone	YKIX716.13					CA17.2A12					P4A10					FJK-16s					YKIX302.9					YCATE55.9				
Company	eBiosciences					AbD Serotec					eBioscience					eBioscience					eBioscience					AbD Serotec				
Volume	5 μl per test (1:20)					8 μl per test (1:12.5)					5 μl per test (1:20)					5 μl per test (1:20)					5 μl per test (1:20)					10 μl per test (1:50)				

*The green-shaded squares indicate the level of brightness for each corresponding fluorochrome, from dim (1 square) to the brightest (5 squares)*.

**Table 2:** Fluorochrome compensation panel graph by sample type and tissue.

**Sample type**	**Marker**						**Blood**	**Lymph node**	**Bone marrow**

	**CD45**	**CD3**	**CD4**	**CD8**	**CD25**	**FoxP3**			
Unstained							X	X	X
Single-stained							X		
							X		
							X		
							X		
							X		
							X	X	X
FMO-CD25							X	X	X
FMO-FoxP3							X	X	X
All							X	X	X

*The green-shaded slots indicate the antibody label used in each sample type, while the crosses indicate which samples were analyzed in each tissue*.

**Figure 3:**
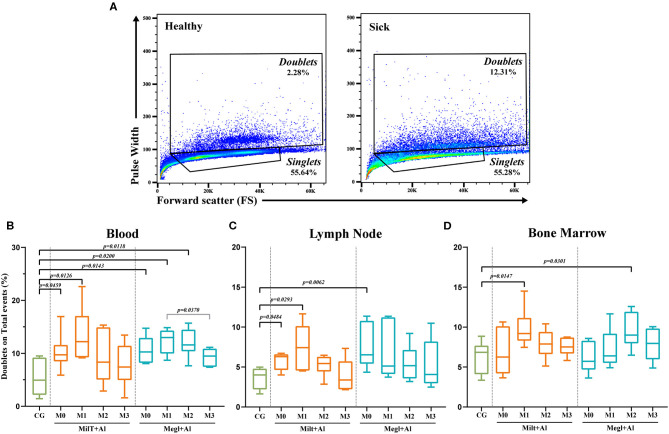
Doublet analysis. **(A)** Gating strategy example in the blood of a healthy [control group (CG)] and a sick dog (M0). Percentage of doublets gated on total events for blood **(B)**, lymph node **(C)**, and bone marrow **(D)** before and after the beginning of treatment. Results of 22 dogs are represented by box and whisker plots and median, minimum, and maximum values. The non-parametric Kruskal–Wallis test (one-way ANOVA on ranks) with Dunn's *post hoc* test was used for statistical comparisons between treatment groups and the control group (CG). The repeated measures ANOVA test with Tukey's *post hoc* test was used for statistical comparisons inside each treatment group. *p*-values are indicated in every statistically significant comparison.

A recent study (67) showed relevant proof that the doublet discrimination usually made in flow cytometry analysis, with the reasoning that they constitute experimental artifacts, may hide cell-to-cell contact, in particular, T cell–monocyte association that is not disrupted during sample processing. Thus, in the current study, a simple approach was used to compare the frequency of doublets in healthy, sick, and treated dogs following the gating strategy shown in Figure 3A.

### Statistical Analysis

Statistical analysis between control, infected, and treated groups was performed using GraphPad Prism software package (version 8.0.1, GraphPad Software Inc.). The Kolmogorov–Smirnoff test was used to assess data normality. The non-parametric Kruskal–Wallis Test (one-way ANOVA on ranks) with Dunn's *post hoc* test was used to evaluate differences in cell subset levels between sick, treated, and control groups. Lastly, the repeated measures ANOVA test with Tukey's *post hoc* test was used to compare dogs between the several months M0, M1, M2, and M3.

## Results

### Canine Leishmaniosis Promotes a High Frequency of Cell Doublets That Reach Healthy Values During Treatment

A significant increase of events in the doublets gate in both blood (*p*_Milt+Al_ = 0.0459; *p*_Megl+Al_ = 0.0143) (Figure 3B) and lymph node (*p*_Milt+Al_ = 0.0484; *p*_Megl+Al_ = 0.0062) (Figure 3C) was observed in sick dogs (M0) when compared with the control group. One month after Milt+Al treatment (M1), blood (*p* = 0.0126), lymph node (*p* = 0.0293), and bone marrow (*p* = 0.0147) presented a significantly high frequency of doublets. Although, during treatment, doublets return to frequencies close to those of the control group. In dogs treated with Megl+Al, peripheral blood exhibited significantly high percentages of doublets in the first (*p*_M1_ = 0.02) and second (*p*_M2_ = 0.0118) months of treatment. On the other hand, the bone marrow presented only a transient increase of doublets 2 months (*p*_M2_ = 0.0301) after the beginning of the treatment (Figure 3D).

### Canine Leishmaniosis Chemotherapy Causes an Imbalance of T Lymphocyte Population

**Figure 4:**
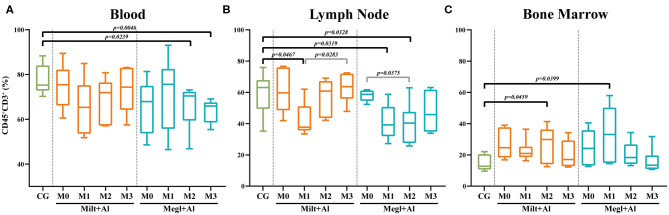
Frequency of lymphocytes (CD45^+^CD3^+^) in the blood **(A)**, lymph node **(B)**, and bone marrow **(C)** of healthy [control group (CG)], sick (M0), and treated dogs (M1, M2, and M3). Results of 22 dogs are represented by box and whisker plots and median, minimum, and maximum values. The non-parametric Kruskal–Wallis test (one-way ANOVA on ranks) with Dunn's *post hoc* test was used for statistical comparisons between treatment groups and the CG. The repeated measures ANOVA test with Tukey's *post hoc* test was used for statistical comparisons inside each treatment group. *p*-values are indicated in every statistically significant comparison.

Peripheral blood (Figure 4A) and lymph node (Figure 4B) of dogs with active leishmaniosis (M0) presented T lymphocyte (CD45^+^CD3^+^) levels similar to clinically healthy dogs. However, the subsequent administration of either treatment resulted in lymphocyte frequency reduction. Dogs under Megl+Al therapy showed a significant reduction of the percentage of blood T cell population (CD45^+^CD3^+^ cells) after 2 (*p*_M2_ = 0.0239) and 3 (*p*_M3_ = 0.0046) months of treatment. However, in the lymph node, a significant frequency reduction of the T cell population was observed at 1 (*p*_M1_ = 0.0319) and 2 (*p*_M2_ = 0.0328) months with this therapy. Furthermore, bone marrow T cells (Figure 4C) frequency significantly increased after the first month of treatment with Megl+Al (*p*_M1_ = 0.0399), reaching values similar to clinically healthy dogs by the second month (M2). One month after the beginning of treatment with Milt+Al, a transient reduction of lymph node T cells (*p*_M1_ = 0.0467) was observed. The bone marrow, in turn, showed a transient higher frequency of T cells (*p*_M2_ = 0.0459) 2 months after treatment, recovering to levels identical to those of control dogs in the third month (M3).

### Anti-leishmanial Therapy Favors the Predominance of CD4^+^ T Cells Over CD8^+^ T Cells

**Figure 5:**
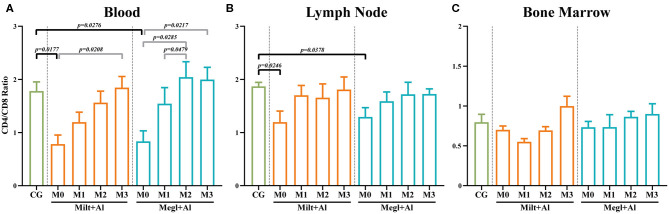
CD4/CD8 ratio in the blood **(A)**, lymph node **(B)**, and bone marrow **(C)** of healthy [control group (CG)], sick (M0), and treated dogs (M1, M2, and M3). Results of 22 dogs are represented by mean values ± SEM. The non-parametric Kruskal–Wallis test (one-way ANOVA on ranks) with Dunn's *post hoc* test was used for statistical comparisons between treatment groups and the CG. The repeated measures ANOVA test with Tukey's *post hoc* test was used for statistical comparisons inside each treatment group. *p*-values are indicated in every statistically significant comparison.

According to several authors, the CD4^+^/CD8^+^ T cell ratio acquired by flow cytometry analysis can be considered a simple and fast way to assess cell-mediated immune response (65, 68). When compared with healthy dogs, blood (*p*_M0_ = 0.0177) (Figure 5A), and lymph node (*p*_M0_ = 0.0246) (Figure 5B) cells of sick dogs presented a significant decrease of the CD4/CD8 ratio to values close to 1, pointing to similar frequencies of CD8^+^ and CD4^+^ T cells. During treatment, this ratio progressed toward values closer to 2, indicating the predomination of CD4^+^ T cells. On the other hand, the bone marrow CD4^+^/CD8^+^ T cell ratio (Figure 5C) of sick dogs was similar to that of healthy dogs, with ratios ranging between 0.5 and 1. These values point toward a variation between a slight predomination of CD8^+^ T cells and an identical frequency of both T cell subsets.

### Canine Leishmaniosis Increases CD4^+^CD8^+^ Double-Positive T Cell Frequency in Peripheral Blood, Lymph Node, and Bone Marrow

**Figure 6:**
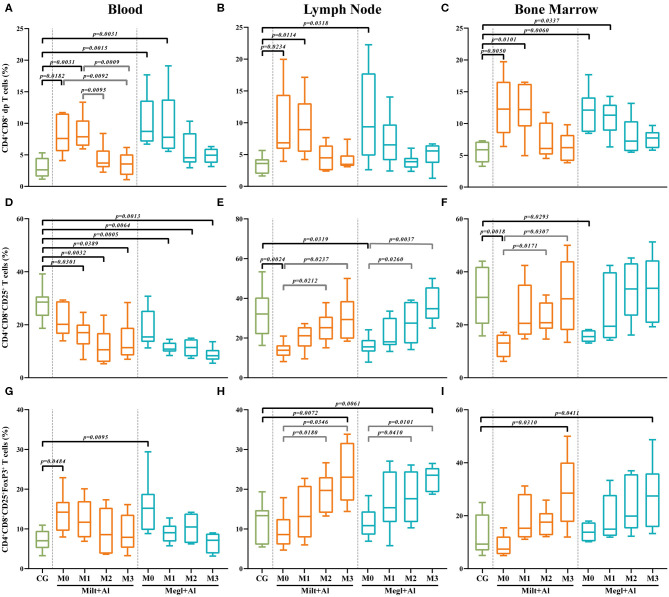
Frequency of CD4^+^CD8^+^ double-positive (dp) T cells. The frequency of dp T cells **(A–C)** expressing CD25 **(D–F)** and CD25 and FoxP3 **(G–I)** was evaluated in the peripheral blood **(A,D,G)**, lymph node **(B,E,H)**, and bone marrow **(C,F,I)** of healthy [control group (CG)], sick (M0), and treated dogs (M1, M2, and M3). Results of 22 dogs are represented by box and whisker plots and median, minimum, and maximum values. The non-parametric Kruskal–Wallis test (one-way ANOVA on ranks) with Dunn's *post hoc* test was used for statistical comparisons between treatment groups and the CG. The repeated measures ANOVA test with Tukey's *post hoc* test was used for statistical comparisons inside each treatment group. *p*-values are indicated in every statistically significant comparison.

Sick dogs (M0) showed increased frequencies of CD4^+^CD8^+^ dp T cells in the blood (Figure 6A) (*p*_Milt+Al_ = 0.0182; *p*_Megl+Al_ = 0.0015), lymph node (Figure 6B) (*p*_Milt+Al_ = 0.0234; *p*_Megl+Al_ = 0.0318), and bone marrow (Figure 6C) (*p*_Milt+Al_ = 0.005; *p*_Megl+Al_ = 0.006) when compared to healthy dogs. The administration of either treatment protocol resulted in a maintenance of these high frequencies of CD4^+^CD8^+^ dp T cells in all tissues during the first month of treatment (M1), progressively normalizing by the following month (M2), with the exception of lymph node of dogs treated with the Megl+Al protocol that recovered 1 month after treatment.

### CD4^+^CD8^+^ Double-Positive T Cells Expressing Regulatory Phenotype Decrease in Peripheral Blood of Sick Dogs and Increase in the Lymph Node and Bone Marrow After Treatment

Lymph node (Figure 6E) and bone marrow (Figure 6F) of sick dogs showed a significant frequency reduction of dp T cells expressing CD25 molecules (lymph node: *p*_Milt+Al_ = 0.0024; *p*_Megl+Al_ = 0.0319/bone marrow: *p*_Milt+Al_ = 0.0018; *p*_Megl+Al_ = 0.0293), which recovered to values similar to clinically healthy dogs during treatment. However, in peripheral blood, treatment caused a significant decrease of this T cell subset (Figure 6D).

In turn, the percentage of CD25^+^FoxP3^+^ dp T cells in the blood of sick dogs (Figure 6G) was higher than that in healthy dogs (*p*_Milt+Al_ = 0.0484; *p*_Megl+Al_ = 0.0095), while being similar to the control group in the lymph node (Figure 6H) and bone marrow (Figure 6I). Treated dogs presented a normalization of the frequencies in the blood after 1 month of treatment, while showing a progressive increase in this subpopulation, reaching higher frequencies than the control group, in the lymph node (*p*_Milt+Al_ = 0.0072; *p*_Megl+Al_ = 0.0061) and bone marrow (*p*_Milt+Al_ = 0.0310; *p*_Megl+Al_ = 0.0411) in the third month.

### *Leishmania* Infection Results in the Increase of Blood CD8^+^ T Cell Frequencies With CD25^+^FoxP3^+^ Phenotype

**Figure 7:**
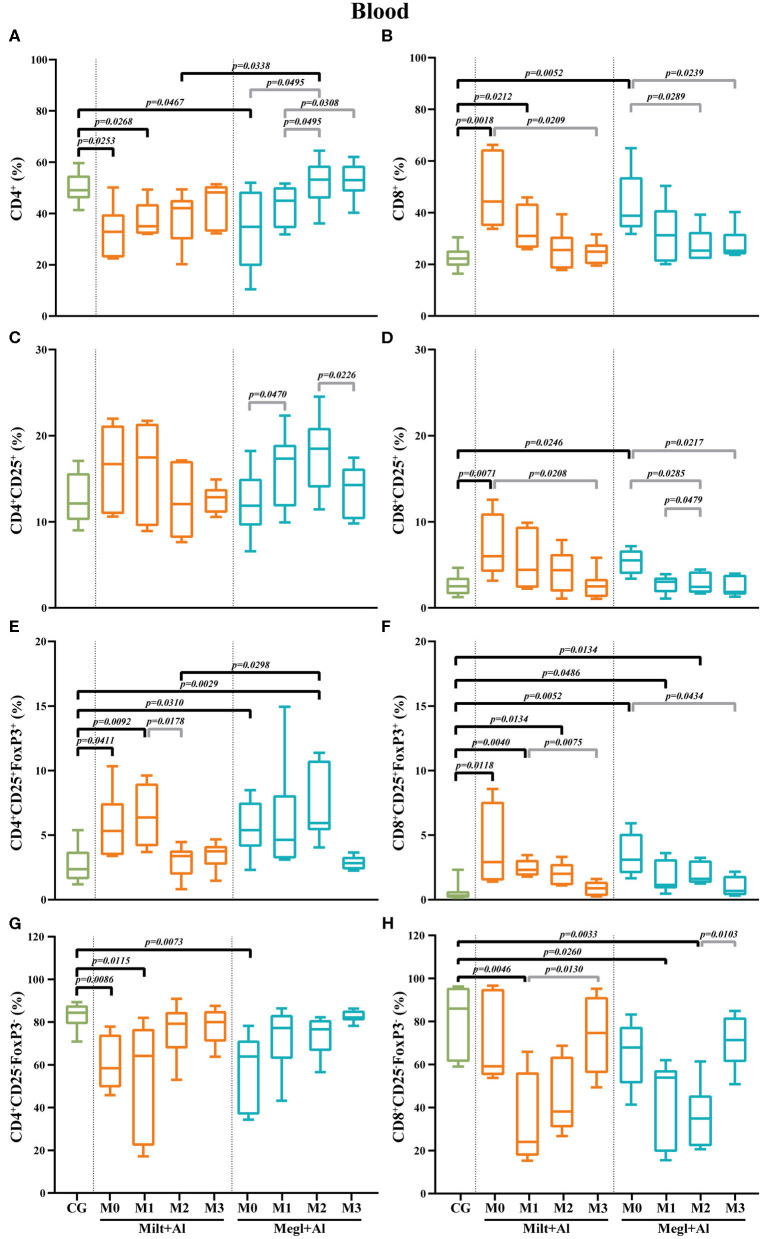
Frequency of CD4^+^ **(A)**, CD8^+^ **(B)**, regulatory (CD25^+^FoxP3^+^) **(C–F)**, and effector (CD4^+^CD25^−^FoxP3^−^/CD8^+^CD25^−^FoxP3^−^) **(G,H)** T lymphocytes in the blood of healthy [control group (CG)], sick (M0), and treated dogs (M1, M2, and M3). Results of 22 dogs are represented by box and whisker plots and median, minimum, and maximum values. The non-parametric Kruskal–Wallis test (one-way ANOVA on ranks) with Dunn's *post hoc* test was used for statistical comparisons between treatment groups and the CG. The repeated measures ANOVA test with Tukey's *post hoc* test was used for statistical comparisons inside each treatment group. *p*-values are indicated in every statistically significant comparison.

Blood of sick dogs (M0) exhibited a significant decrease in the frequency of the CD4^+^ T cell subset (*p*_Milt+Al_ = 0.0253; *p*_Megl+Al_ = 0.0467) (Figure 7A) along with a high frequency of the CD8^+^ T cell subset (*p*_Milt+Al_ = 0.0018; *p*_Megl+Al_ = 0.0052) (Figure 7B). Both treatments were able to recover normality for the CD4^+^ and CD8^+^ T cell fractions. However, dogs under the Megl+Al protocol recovered to values similar to those of clinically healthy dogs during the first month of treatment (M1), faster than the group treated with Milt+Al that only recovered after the second month (M2).

The frequency of blood T cells with CD4^+^CD25^+^ phenotype showed some fluctuation, mainly during Megl+Al treatment (Figure 7C), although with no statistical differences when compared with clinically healthy dogs. However, a significant increase in the frequency of the CD8^+^CD25^+^ T cell subset (*p*_Milt+Al_ = 0.0071; *p*_Megl+Al_ = 0.0246) was observed in sick dogs (M0) when compared with that of the control group (Figure 7D). This cell subset returned to normal values immediately after the beginning of both treatments (M1).

CD4^+^CD25^+^FoxP3^+^ (*p*_Milt+Al_ = 0.0411; *p*_Megl+Al_ = 0.0310) and CD8^+^CD25^+^FoxP3^+^ (*p*_Milt+Al_ = 0.0118; *p*_Megl+Al_ = 0.0052) T cell subsets of sick dogs (M0) presented higher frequencies than those of the control group (Figures 7E,F). After administration of both treatments, an increase in the frequency of the CD4^+^CD25^+^FoxP3^+^ T cell subset was observed (*p*_Milt+Al(M1)_ = 0.0092; *p*_Megl+Al(M2)_ = 0.0029), with the values returning to healthy levels at M2 and M3, for the Milt+Al and Megl+Al groups, respectively. Likewise, the CD8^+^CD25^+^FoxP3^+^ T cell subset recovered to values comparable to those of control dogs after 3 months for both treatment protocols.

Effector T cell subsets of sick dogs (M0) presented different patterns. CD4^+^CD25^−^FoxP3^−^ T cells were significantly lower than those of the control group (*p*_Milt+Al_ = 0.0086; *p*_Megl+Al_ = 0.0073). However, dogs recovered to healthy values 1 month after the beginning of treatment with Megl+Al (M1) and after 2 months of Milt+Al therapy (M2) (Figure 7G). On the other hand, CD8^+^CD25^−^FoxP3^−^ T cells of sick dogs were similar to those of healthy dogs, but subsequent treatments led to a significant reduction in cell frequency (*p*_Milt+Al(M1)_ = 0.0046; *p*_Megl+Al(M1)_ = 0.026), with the Megl+Al group recovering to normal frequencies by the third month (M3) and the Milt+Al group after the second month (M2) (Figure 7H).

### Canine Leishmaniosis Promotes the Increase of Lymph Node CD8^+^ T Cell Frequencies, and Treatment Leads to an Imbalance of Effector and Regulatory T Cell Subsets

**Figure 8:**
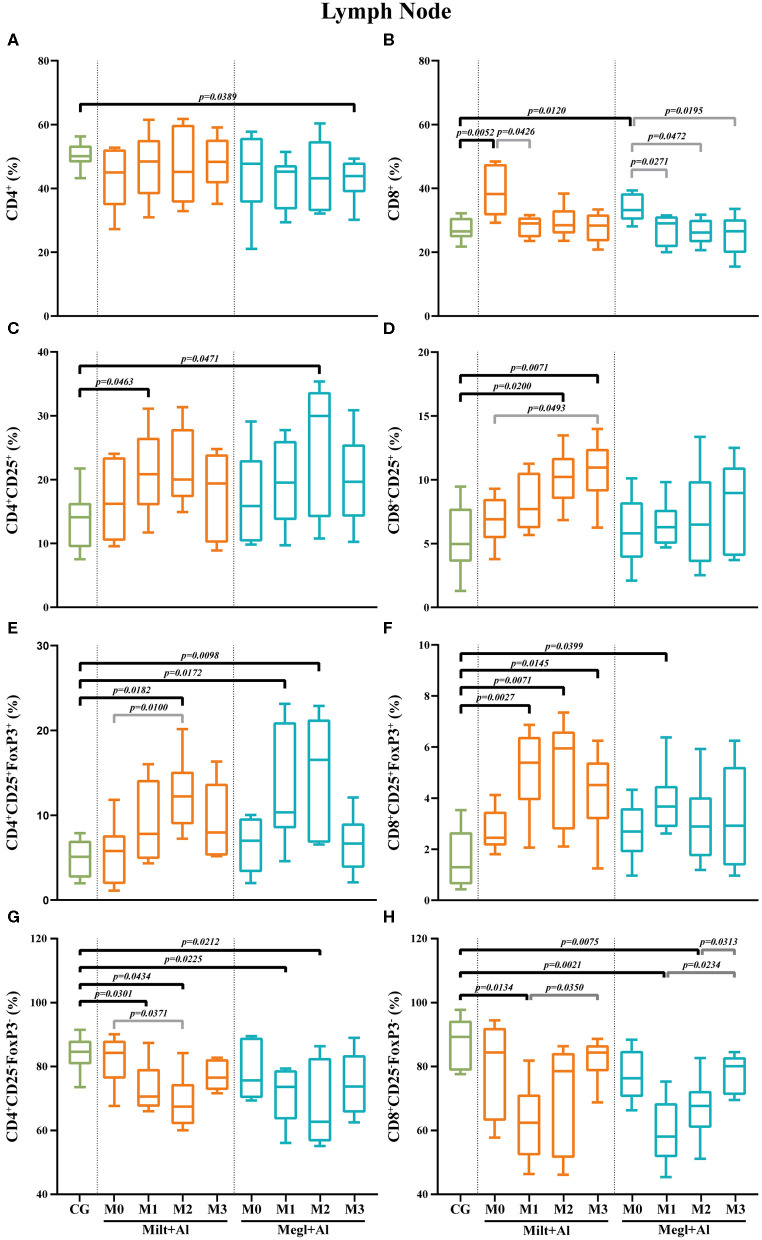
Frequency of CD4^+^ **(A)**, CD8^+^ **(B)**, regulatory (CD25^+^FoxP3^+^) **(C–F)**, and effector (CD4^+^CD25^−^FoxP3^−^/CD8^+^CD25^−^FoxP3^−^) **(G,H)** T lymphocytes in the lymph node of healthy [control group (CG)], sick (M0), and treated dogs (M1, M2, and M3). Results of 22 dogs are represented by box and whisker plots and median, minimum, and maximum values. The non-parametric Kruskal–Wallis test (one-way ANOVA on ranks) with Dunn's *post hoc* test was used for statistical comparisons between treatment groups and the CG. The repeated measures ANOVA test with Tukey's *post hoc* test was used for statistical comparisons inside each treatment group. *p*-values are indicated in every statistically significant comparison.

In the lymph node of sick dogs, the frequency of CD4^+^ T cells was similar to that of healthy dogs (Figure 8A), but the CD8^+^ T cell fraction presented a higher percentage (*p*_Milt+Al_ = 0.0052; *p*_Megl+Al_ = 0.0120) (Figure 8B). Furthermore, treatment administration caused a reduction of the CD8^+^ T cell frequencies to values similar to control dogs. Three months after the onset of treatment with Megl+Al, the CD4^+^ T cell fraction was significantly diminished (*p* = 0.0389) when compared with clinically healthy dogs.

In sick dogs, the level of CD4^+^ (Figure 8C) and CD8^+^ (Figure 8D) T cells with CD25^+^ phenotype was similar to healthy dogs. However, both treatment protocols led to a transient increase of the CD4^+^CD25^+^ T cell subset frequencies after 1 month of Milt+Al treatment (*p* = 0.0463) and 2 months of Megl+Al (*p* = 0.0471). The CD8^+^CD25^+^ T cell subpopulation of dogs under the Milt+Al protocol showed a significant increase 2 (*p* = 0.0200) and 3 (*p* = 0.0071) months after the beginning of treatment (Figure 8D).

Likewise, sick dogs showed similar frequencies of CD4^+^CD25^+^FoxP3^+^ and CD8^+^CD25^+^FoxP3^+^ T cells compared to healthy dogs. Moreover, after treatment, these dogs exhibited a significant increase in the frequency of the CD4^+^CD25^+^FoxP3^+^ T cell subset (Figure 8E). In dogs treated with Milt+Al, a peak of the frequency of CD4^+^ Treg cells was observed 2 months (*p*_M2_ = 0.0182) after the beginning of treatment. One and 2 months after administration, Megl+Al also promoted a CD4^+^ Treg frequency increase (*p*_M1_ = 0.0172; *p*_M2_ = 0.0098) that subsequently reverted to normal values. Moreover, Milt+Al caused a significant increase in the frequency of CD8^+^CD25^+^FoxP3^+^ T cells (*p*_M1_ = 0.0027; *p*_M2_ = 0.0071; *p*_M3_ = 0.0145), while the Megl+Al protocol only resulted in a transient increase of this subpopulation 1 month after treatment (*p*_M1_ = 0.0399) (Figure 8F).

Effector T cell subsets in the lymph node of sick dogs were similar to those of healthy dogs. After treatment administration, CD4^+^CD25^−^FoxP3^−^ T cell frequencies showed a progressive reduction during the first and second month with both the Milt+Al (*p*_M1_ = 0.0301; *p*_M2_ = 0.0434) and the Megl+Al protocol (*p*_M1_ = 0.0225; *p*_M2_ = 0.0212) (Figure 8G). CD8^+^CD25^−^FoxP3^−^ T cell frequencies also presented a significant reduction after drug administration (*p*_Milt+Al_ = 0.0134; *p*_Megl+Al_ = 0.0021), with the Milt+Al-treated dogs recovering cell frequency levels by the second month (M2) and the Megl+Al-treated dogs by the third month (M3) (Figure 8H).

### *Leishmania* Infection Causes the Increase of Bone Marrow CD8^+^ T Cell Frequencies With CD25^+^FoxP3^+^ Phenotype

**Figure 9:**
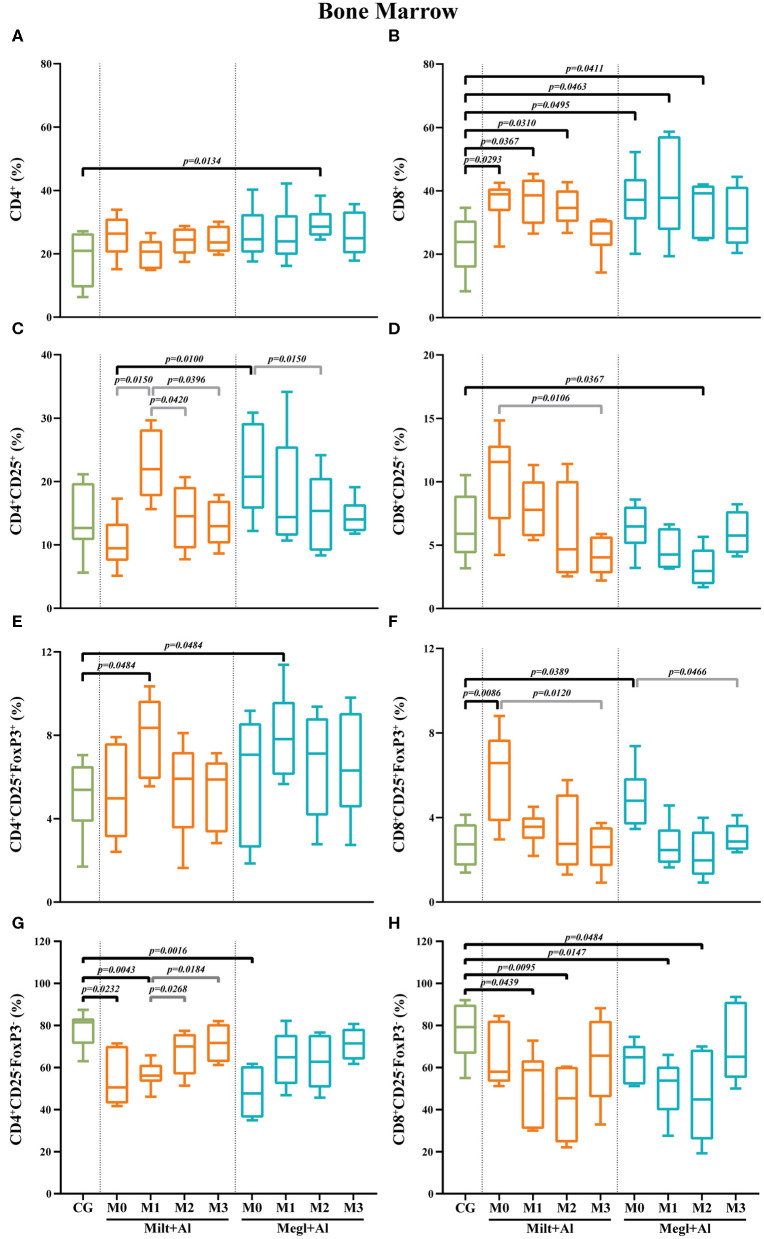
Frequency of CD4^+^ **(A)**, CD8^+^ **(B)**, regulatory (CD25^+^FoxP3^+^) **(C–F)**, and effector (CD4^+^CD25^−^FoxP3^−^/CD8^+^CD25^−^FoxP3^−^) **(G,H)** T lymphocytes in the bone marrow of healthy [control group (CG)], sick (M0), and treated dogs (M1, M2, and M3). Results of 22 dogs are represented by box and whisker plots and median, minimum, and maximum values. The non-parametric Kruskal–Wallis test (one-way ANOVA on ranks) with Dunn's *post hoc* test was used for statistical comparisons between treatment groups and the CG. The repeated measures ANOVA test with Tukey's *post hoc* test was used for statistical comparisons inside each treatment group. *p*-values are indicated in every statistically significant comparison.

In the bone marrow of sick dogs, the frequency of CD4^+^ T cells (Figure 9A) was similar to clinically healthy dogs. The administration of Milt+Al did not cause significant alterations in the CD4^+^ T cell fraction, while dogs under the Megl+Al protocol exhibited a transient frequency increase (*p* = 0.0134) 2 months after the onset of treatment. Meanwhile, a prominent increase of the frequency of CD8^+^ T cells was observed in sick dogs (*p*_Milt+Al_ = 0.0293; *p*_Megl+Al_ = 0.0495) (Figure 9B). This high frequency of CD8^+^ T cells in the bone marrow persisted during both treatments (Milt+Al: *p*_M1_ = 0.0367; *p*_M2_ = 0.0310) (Megl+Al: *p*_M1_ = 0.0463; *p*_M2_ = 0.0411), returning to values similar to control dogs by the third month (M3).

Regarding the CD4^+^CD25^+^ T cell subpopulation (Figure 9C), no considerable differences were observed in the bone marrow of sick dogs when compared with that of clinically healthy dogs. Moreover, dogs treated with Megl+Al evidenced a transient decrease of the frequency of CD8^+^CD25^+^ T cells by month 2 (*p*_M2_ = 0.0367) that quickly recovered (Figure 9D).

In the bone marrow of sick dogs, the frequency of Treg cells (CD4^+^CD25^+^FoxP3^+^) was similar to that of control dogs (Figure 9E). Nevertheless, an increase of the frequency of this cell subset was observed 1 month (*p*_Milt+Al_ = 0.0484; *p*_Megl+Al_ = 0.0484) after either treatment, followed by normalization. Similar to peripheral blood, the CD8^+^CD25^+^FoxP3^+^ T cell subset frequencies (Figure 9F) of sick dogs was significantly higher (*p*_Milt+Al_ = 0.0086; *p*_Megl+Al_ = 0.0389). Both treatments led to a reduction of cell frequencies to values similar to those of the control group.

CD4^+^CD25^−^FoxP3^−^ T cell frequencies of sick dogs were significantly lower in comparison with those of healthy dogs (*p*_Milt+Al_ = 0.0232; *p*_Megl+Al_ = 0.0016) (Figure 9G). However, the Megl+Al group recovered to values close to those of healthy dogs 1 month earlier than the Milt+Al group. The frequencies of CD8^+^CD25^−^FoxP3^−^ T cells of sick dogs, on the other hand, were similar to those of healthy dogs, with the administration of either treatment leading to a significant decrease 1 (*p*_Milt+Al_ = 0.0439; *p*_Megl+Al_ = 0.0147) and 2 months (*p*_Milt+Al_ = 0.0095; *p*_Megl+Al_ = 0.0484) after the beginning of treatment (Figure 9H).

## Discussion

CanL treatment has an inherent connection with the ability of the dog's immune system to develop a competent cellular immune response against *L. infantum*. Thus, comprehending the cellular immune response and the dynamics of T cell subsets in dogs naturally infected with *Leishmania*, especially in organs that usually harbor these parasites, is of utmost relevance not only for the treatment and management of CanL but also as guidelines for the development of prophylactic and therapeutic tools. A better knowledge of the effect of antileishmanial therapy on the cellular immune response of dogs can facilitate the development of strategies to reduce the transmission of the parasite and, consequently, lead to a decrease in the incidence of zoonotic visceral leishmaniosis. Therefore, in the current study, T cell subpopulations of dogs naturally infected with *L. infantum* were phenotypically characterized before treatment and during the influence of antileishmanial drugs.

In the current study, it was found that sick dogs have increased doublet frequencies in peripheral blood and lymph node, decreasing to values similar to clinically healthy dogs after treatment. As was proposed by Burel et al. (67), these changes in the doublet levels associated with CanL and during the first months of treatment may reflect a possible cell-to-cell interaction between T lymphocytes and antigen-presenting cells. It is also possible that the doublets could increase as a result of interaction of Treg:lymphocyte, as Treg cells, which seem to be increased in CanL, appear to exert immune suppression by mechanisms dependent on cell contact (69). In the present work, it was not possible to delve deeper into these interactions since this was a secondary objective of the study. In this sense, not enough events were collected in the doublet region to obtain meaningful information on further subpopulations. This way, further detailed studies are needed to corroborate this hypothesis, with the correlation between CanL and the level of doublets being able to be used as a possible marker of disease to monitor treatment success and predict potential relapses (67).

Several authors have correlated symptomatic dogs with decreased levels of CD4^+^ T cells and CD4/CD8 ratios in peripheral blood (35, 70), along with high antibody titers. Other authors verified that higher infectivity to sand flies by naturally infected dogs was associated with lower proportions of CD4^+^ T cells in the blood (37). Furthermore, it has also been shown that the administration to dogs infected with *Leishmania* of antileishmanial drugs, such as amphotericin B and meglumine antimoniate, promoted the increase of the percentage and the absolute cell count of CD4^+^ T cells in the blood, respectively (36, 71). On the other hand, other treatment protocols, such as allopurinol in monotherapy, although able to improve the number of circulating CD4^+^ T cells in the blood, were not able to restore values to those within the normal range (68). Thus, the findings obtained in the current study are in line with previous reports. Sick dogs presented low CD4/CD8 ratios in peripheral blood and lymph node, recovering to values equal to the healthy group after the administration of both treatments. Following our results, and according to several authors (36–38, 68, 71), the CD4/CD8 ratio can be a useful indicator of the immunological condition of sick dogs and a possible tool with prognostic value. Some authors also describe a decline of the percentage of CD3^+^ lymphocytes in the peripheral blood of CanL symptomatic dogs, as a direct consequence of the reduction in the frequency of CD4^+^ T cells (36, 56). Other authors, on the contrary, have reported a significant increase of CD3^+^ T cells in sick dogs, especially in dogs severely affected (72). Nevertheless, the administration of antileishmanial therapy in both situations restored CD3^+^ lymphocytes within normal values (36, 56, 72). Moreover, the results of the present study point to a dual effect of antileishmanial therapy on bone marrow and lymph node. Both treatments led to a reduction in the frequency of lymph node T cells (CD45^+^CD3^+^) along with an increase in the bone marrow. Interestingly, only meglumine antimoniate in association with allopurinol resulted in a decrease of the frequency of blood T cells.

Protective immunity against CanL is usually considered to be dependent on a Th1 immune response (6). The predominance of IFN-γ-producing CD4^+^ T cells is crucial for macrophage activation in order to kill internalized *Leishmania* through the production of NO and ROS (73, 74). A reduction of the CD4^+^ T cell population is usually associated with the inability to control the infection, allowing the survival and replication of *Leishmania* parasites in macrophages, which can subsequently lead to increased infectibility to sand flies (37). Murine studies have shown that *Leishmania* parasites negatively interfere with the ability of IFN-γ to induce the expression of MHC-II mRNA, leading to parasitized macrophages with a low expression of MHC class II molecules (75). Thus, due to their reduced capacity as antigen-presenting cells, these macrophages are therefore unable to provide co-stimulatory signals to CD4^+^ T cells (76, 77), which, in turn, are not stimulated, do not proliferate, and do not produce IFN-γ. Although the complete role of CD8^+^ T cells in CanL is still debated, there are studies of leishmaniosis in humans and mice showing a functional duality. CD8^+^ T cells can either play a protective role by releasing IFN-γ, or they can be pathogenic to the host, causing excessive inflammation at the site of infection (73) as a result of cytotoxic activity, which can exacerbate disease progression (78). Following the results of previous reports (35, 36, 79), the sick dogs included in the current study also showed an increased frequency of CD8^+^ T cells in the blood, lymph node, and bone marrow, along with significantly decreased levels of CD4^+^ T cells in the blood. These findings suggest that CD8^+^ T cells are at the forefront of the fight against *Leishmania* infection, especially in tissues that commonly harbor *Leishmania* parasites. Nonetheless, antileishmanial therapy led to the recovery of the T cell population in all tissues. And whether due to the direct action of the antileishmanial drugs or the availability of free antigens as a consequence of *Leishmania*'s death caused by therapy, a shift of T cell population occurs, leading to a rapid reduction in the frequency of CD8^+^ T cells in the blood and lymph node.

Regulatory T cells are generally considered to be a subset of CD4^+^ T cells, which express the non-constitutive IL-2R-α chain (CD25) and the transcriptional factor FoxP3 (80, 81). The main function of these cells is to suppress excessive or misguided immune responses and prevent autoimmune diseases (74, 82). Few are the Treg studies done in CanL, which account for the lack of overall information on these subpopulations (83). In dogs experimentally infected with *L. infantum*, FoxP3 RNA was increased in the skin and liver, but in the lymph node, the authors verified a decrease associated with disease progression (84). Figueiredo et al. (85) referred that CanL enhanced FoxP3 expression in the jejunum and colon. However, the skin of *L. chagasi* (syn. *L. infantum*)-infected dogs revealed lower levels of FoxP3 expression (86). Another study found no correlation between TGF-β or IL-10 producing CD4^+^ Treg cells in the blood and spleen and the parasitic load of naturally infected dogs (87).

In the present study, sick dogs showed increased frequencies of blood CD4^+^ Treg cell associated with decreased percentages of CD4^+^ (CD25^−^FoxP3^−^) effector T cells, signaling a lack of adequate cellular immune response, which can prolong the presence of the parasite, facilitating parasite transmission. Antileishmanial therapy allowed the normalization of blood CD4^+^ Treg and effector T cell subsets, especially in dogs under the meglumine plus allopurinol protocol, restoring the action of CD4^+^ effector T cells.

Curiously, and following the obtained results, CanL does not seem to cause significant changes in CD4^+^ Treg cells and CD4^+^ effector T cell subsets of lymph nodes. Similarly, in a study with mice infected with *L. infantum*, a high frequency of CD4^+^CD25^+^ T cells expressing FoxP3 was found in the lymph nodes in the first weeks of infection, followed by a decrease in the subsequent chronic phase of the disease (43), supporting the observed results in the present study. In addition, the administration of CanL drugs caused a transient disturbance in Treg cells and effector T cell subsets. By directing the reduction in the frequency of effector T cells associated with the increase of the Treg cell subset, therapy appears to promote the development of a suppressive immune response located in the dog's lymph node. Despite this, 3 months after the start of treatment, the values normalize. Therefore, it is possible that miltefosine and meglumine antimoniate, which were administered to sick dogs only during the first 4 weeks of treatment, are primarily responsible for the development of a suppressive immune response that can limit inflammation.

In patients with visceral leishmaniosis caused by *L. donovani*, the bone marrow revealed an increase of Treg cells (CD4^+^CD25^+^FoxP3^+^) that outnumbered effector T cells (CD4^+^CD25^+^FoxP3^−^) (42). These Treg cells were shown to be a source of IL-10 and persisted in patients even after successful chemotherapy with sodium antimony gluconate. In the current study, both treatments induced a quick increase in the frequency of the CD4^+^CD25^+^FoxP3^+^ and CD4^+^CD25^−^FoxP3^−^ T cell subsets in the bone marrow of dogs, but for a short period of time, normalizing by the second month of observation. In this case, the findings support the hypothesis that the increase in the frequency of CD4^+^ Treg cells can be a possible consequence of miltefosine and meglumine antimonial drugs.

In CanL, as in other diseases in which the immune system is deeply involved, the presence and action of CD8^+^ Treg cells are still a matter of discussion. In a study of human visceral leishmaniosis, the authors proposed that IL-10 produced by CD8^+^ T cells could lead to a downregulation of cytokine production, in particular pro-inflammatory cytokines like TNF-α and IFN-γ, blocking this way the anti-leishmanial macrophage activity (88). Subsequent studies have shown the presence of a subset of CD8^+^ Treg cells that can inhibit the CD4^+^ T cell-mediated immune response by inducing apoptosis of activated CD4^+^ T cells (89). This way, the increased frequency of the CD8^+^CD25^+^FoxP3^+^ T cell subset in the blood and bone marrow of sick dogs shown in the current study could represent a complementary mechanism of immune regulation that may favor parasite survival (78). Treatment of CanL with miltefosine or meglumine antimoniate in combination with allopurinol directs blood CD8^+^ Treg cells to progressively return to normal values. These antileishmanial drugs seem to cause a shift in blood and bone marrow lymphocytes by reducing the increased frequency of the CD8^+^CD25^+^FoxP3^+^ T cell subset and reduce effector CD8^+^ (CD25^−^FoxP3^−^) T cells to restrain the local inflammatory immune response and cytotoxicity in order to lessen possible tissue damage.

CD4^+^CD8^+^ dp T cells have been identified in dogs with and without CanL (56, 90, 91). In the current study, the frequency of CD4^+^CD8^+^ dp T cell subsets was revealed to be increased in peripheral blood, lymph node, and bone marrow of dogs with CanL. Considering the chronic profile of CanL, these findings are in line with previous studies (57–61) that have established a link between increased dp T cells and chronic diseases. Furthermore, dp T cells have also been associated with increased production of IFN-γ in pigs (50), similar to previous results found in dogs with CanL (66). Moreover, the presence of CD4^+^CD8^+^CD25^+^FoxP3^+^ T cell subset in the peripheral blood of sick dogs reveals a possible regulatory activity, as proposed by other authors (62), while the lymph node and bone marrow presented decreased percentages of CD25, reflecting a possible cytotoxic role (63) resulting from the infection with *L. infantum*. In turn, in the present study, the administration of either treatment led to a change in both profiles, with dp T cells in the blood losing the regulatory phenotype, possibly in order to fight the infection, while the lymph node and bone marrow apparently switching to a regulatory profile to nullify a possible excessive cytotoxic damage. In any case, since the role of these CD4^+^CD8^+^ dp T cells is not yet fully understood *in vivo*, further in-depth studies are still needed in these subpopulations in order to elucidate their modes of action.

The immune response to *Leishmania*, in humans, mice, or dogs, seems to be far complex and influenced by several types of immune cells and different immune mediators, establishing an elaborate network. Either way, there seems to be a consensus that *Leishmania* parasites lead to differentiation of specific cell immunophenotypes in different tissues. CanL in this study led to an increased frequency of CD8^+^ T cells in all tissues, along with increased CD4^+^CD8^+^ dp T cell frequencies, resulting in a predominant pro-inflammatory profile. CD8^+^ Treg cell frequencies were also significantly increased in the blood and bone marrow, showing a possible action on immune responses mediated by CD4^+^ T cells, which can lead to parasite tolerance and disease progression. In the present work, the administration of either treatment protocol led to an overall recovery of the T cell subpopulations by the end of observation, reflecting the clinical improvement of the dogs (66). Nonetheless, it should be noted that both protocols resulted in an increase of CD4^+^ Treg cell frequencies in all tissues, possibly in order to significantly reduce the frequency of CD8^+^CD25^−^FoxP3^−^ T cells present and to control the local inflammatory immune responses. Lastly, with respect to the effectiveness of either treatment, despite not being the scope of this work, the recovery of many subpopulations was achieved more quickly with the Megl+Al protocol than with the Milt+Al protocol, which is in agreement with previous results (66).

Monitoring T cell subsets by using specific biomarkers and analyzing the effectiveness of CanL treatments allow a better understanding of the interplay between the parasite and the dog's immune response, which should improve patient management, lead to the development of more efficient and less toxic chemotherapies, and encourage the use of prophylactic measures that favor the reduction of zoonotic visceral leishmaniasis.

## Data Availability Statement

The datasets generated for this study are available on request to the corresponding author.

## Ethics Statement

The animal study was reviewed and approved by Ethics and Animal Welfare Committee of the Faculty of Veterinary Medicine, University of Lisbon. Written informed consent was obtained from the owners for the participation of their animals in this study.

## Author Contributions

GS-G, IP, and MS conceived and designed the study. AB, AR, IP, JM, MP, and MS collected samples. IP, LG, and MS processed samples and did subsequent microscopic, serological, and molecular tests. GA-P and MS conducted the experiments. GA-P, GS-G, IP, and MS analyzed the data. GS-G and MS conducted statistical analysis. GS-G, IP, and MS drafted the manuscript. AVR, GA-P, GS-G, and IP made in-depth reviews of the manuscript. All authors read and approved the final manuscript.

## Conflict of Interest

The authors declare that the research was conducted in the absence of any commercial or financial relationships that could be construed as a potential conflict of interest.

## Abbreviations

ALT: alanine aminotransferase
AST: aspartate aminotransferase
BID: bis in die
BUN: blood urea nitrogen
CanL: canine leishmaniosis
CD: cluster of differentiation
CG: control group
CVBD: canine vector-borne disease
DNA: deoxyribonucleic acid
FMO: fluorescence minus one
FoxP3: forkhead box protein 3
IFN-γ: interferon gamma
IL-2: interleukin 2
IL-10: interleukin 10
Megl+Al: meglumine antimoniate with allopurinol
Milt+Al: miltefosine with allopurinol
SID: semel in die
TGF-β: transforming growth factor beta
Th1: type-1 T-helper
Th2: type-2 T-helper
TNF-α: tumor necrosis factor alpha
Treg cells: regulatory T cells
UPC: urine protein-to-creatinine ratio.
